# Impact of a virtual nurse-led Early paLlIative Care IntervenTion (ELICIT) randomized controlled trial

**DOI:** 10.1038/s41746-026-02935-5

**Published:** 2026-06-25

**Authors:** Vyjeyanthi S. Periyakoil, Eric Neri, Kristine Isabel M. Mallari, Hsiao Min Huang, Elizabeth Deleon Padilla, Kenneth Covinsky, Charles F. von Gunten, Helena Kraemer

**Affiliations:** 1 Stanford University, Division of Primary Care and Population Health, Department of Medicine, School of Medicine, Palo Alto, CA USA; 2 Veterans Affairs Palo Alto Health Care System, Palo Alto, CA USA; 3 UCSF, San Francisco, CA USA; 4 UCSD, San Diego, CA USA

**Keywords:** Health care, Medical research

## Abstract

In this 12-month, virtual, RCT, we compared usual care to a nurse-led primary palliative care intervention on chronically ill, community-dwelling older adults across the United States with varying levels of cognitive impairment. Significantly higher rates of advance care planning (89.1% vs 3.0%) and reporting of supportive care needs (86.1% vs 29.3%) in the intervention vs usual care arm highlights the improvement in the care of older adults with cognitive impairment. *Trial Registration:* https://clinicaltrials.gov/study/NCT03881579.

Dementia, a serious illness, was the top hospice diagnosis in the US in 2023 (NHPCO, 2024). About 6.7 million older Americans are currently living with dementia, and this number is projected to double by the year 2060^1^. Four out of five people living with dementia (PLWD) live in the community (ASPE, 2017), and many live alone.

There is an urgent need for innovative and scalable models to facilitate early primary palliative care for all older adults with chronic illness who are at risk for cognitive impairment, as it improves care^2^. Experts have identified that the four domains (WHO, 2012) of primary palliative care are as follows : (a) assessing and palliating physical symptoms, (b) assessing and managing psychological, social, cultural, and spiritual aspects of care, (c) establishing goals of care, identification of a surrogate decision maker and completion of advance care planning documentation; and (d) providing care coordination support. Nurses provided primary palliative care for the persons randomized to the intervention arm.

Currently, hospitalized patients and hospice dementia patients^3^ are more likely to have access to palliative care, and research has focused predominantly on institutionalized dementia patients in the last phase of life. However, patients report primary palliative care needs at or before the time of diagnosis and across the early trajectory of cognitive impairment^4^. Although experts^5,6^ have emphasized that primary palliative care is a fundamental aspect of high-quality patient care, community-dwelling PLWD do not have access due to the shortage of trained personnel^7^. As a result, PLWD struggle with undiagnosed and untreated physical, emotional and existential symptoms^4,8^ for many years prior to death.

Utilizing trained nurses to provide primary palliative care to chronically ill older adults living in the community will fill a large and unmet need for these services and facilitate timely access to primary palliative care for these patients. Most of the palliative care in home hospice is currently provided by registered nurses (RNs). Hospice nurses (*Bureau of Labor Statistics, 2015*) monitor patients, assess their pain and other symptoms, serve as case managers and patient advocates, and provide education and support to the patients and their family members. Currently, registered nurses do not provide primary palliative care for seriously ill patients who are in the earlier stages of their illness trajectories, even though they can be trained to do so. Evidence about the feasibility and scope of practice of a nurse-led primary palliative care model for community-dwelling patients is also sparse^9^.

This randomized clinical trial (RCT) was designed to compare the impact of {usual care} to {usual care plus a virtual, nurse-led, primary palliative care intervention} on community- dwelling, chronically ill older adults with varying degrees of cognitive impairment. Control Condition {Usual Care}: Participants randomized to the usual care (UC) arm received care from their primary care provider (PCP) and their care team. In this case, usual care refers to the standard or routine out-patient care that participants receive from their primary care team. This included any appointments, consultations and procedures that are standard care for out-patient primary care. Intervention Arm (EPC): Participants in the intervention arm received {Usual care + virtual, nurse-led early primary palliative care intervention}. We hypothesized that a virtual, nurse-led primary palliative care intervention will (a) facilitate timely goals-of-care conversations and advance care planning and (b) result in better assessment, and care coordination of the patients’ primary palliative needs. Our protocol is available online^10^.

200 community-dwelling older adults (–) with a Clinical Dementia Rating scores^11^ of 0, 0.5, 1 or 2 were enrolled. Out of the 200, 189 (94.5%) patients received care from other healthcare systems and 11 received care at Stanford. At the time of study entry, all participants had high (94.5%) or moderate (5.5%) comorbidity as measured by the Charlson Comorbidity Index. The standard score cutoffs are: 0–2 (Low/Mild), 3–4 (Moderate), and ≥5 (High/Severe)^12^. 101 were randomized to the intervention and 99 to usual care. Figure 1 shows the CONSORT diagram and Table 1 shows participant characteristics. For patients allocated to the intervention arm, the intervention included: goals-of-care discussion with a nurse, completion and signing of both Advance Directives (Stanford Letter Project, 2026) and Physician’s Orders for Life Sustaining Treatment (POLST,2026) forms, and uploading these documents into the electronic health record, with the original copies returned to patients for their records. During the study, 89.1% (90 of 101) of the patients allocated to the intervention arm completed goals-of-care discussions, and documented AD and POLST compared to only 3.0% (3 of 99) of the patients allocated to the control arm (Fig. 2). The differences between the two arms were highly significant (*p* value < 0.001), with a large effect size (AUC = 0.99 and SRD = 0.97). For the patients (*n* = 101) in the intervention arm, the nurses worked with 88 individual primary care doctors. According to the participants’ report, during the clinic appointment, their doctor verified the patient’s goals-of-care, reviewed and finalized the POLST form and signed it with the patient. Within the intervention arm, on average, patients with a baseline CDR > 0 took longer to complete the AD and POLST compared to those with a baseline CDR = 0 (see Fig. 2).

**Fig. 1:**
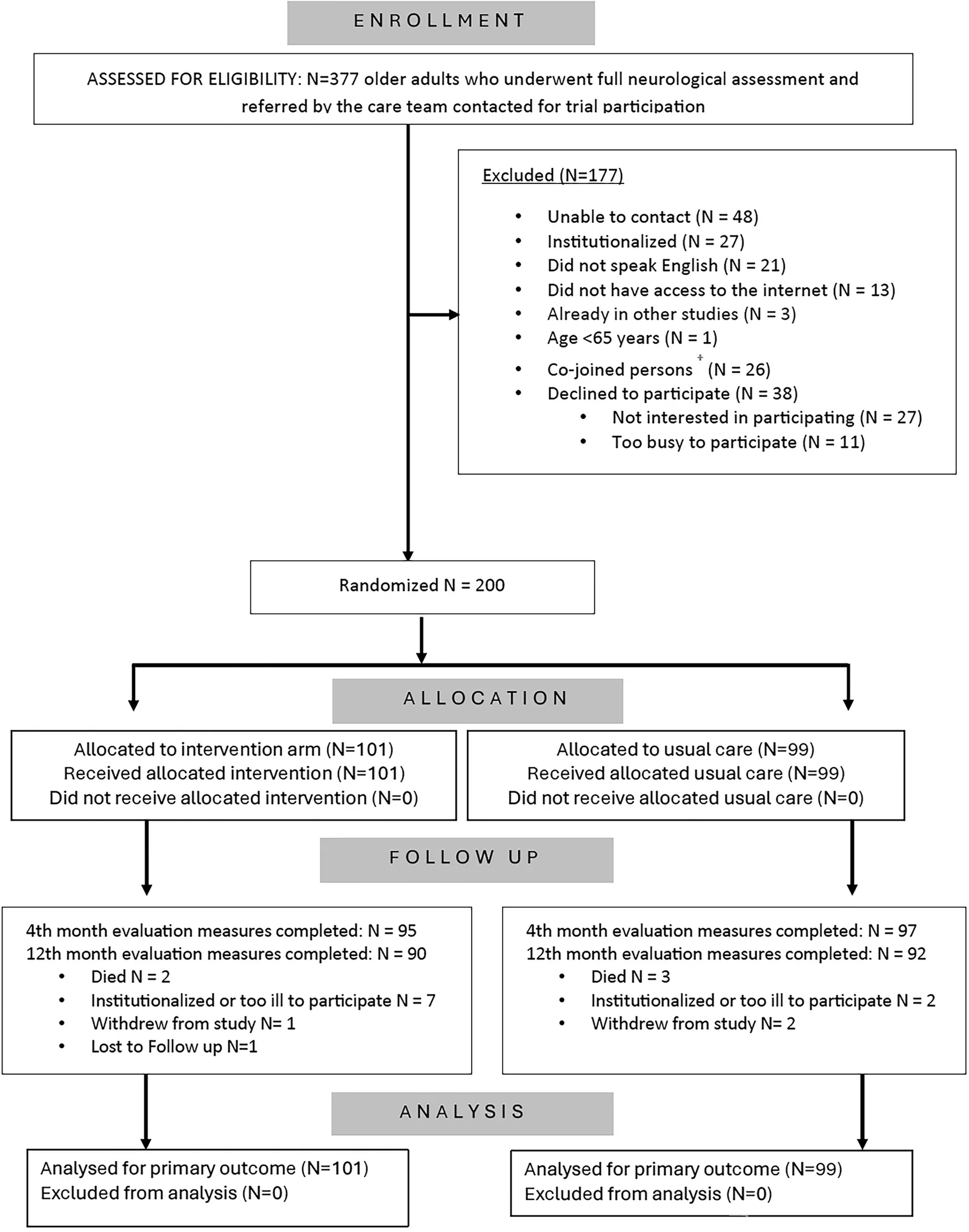
CONSORT flow diagram of the progress through the phases of the ELICIT RCT (enrollment, intervention allocation, follow-up, and data analysis). + Cojoined persons = When a couple living in the same household wanted to participate in the study, only one was entered into the study and randomized. The other person was given access to the same treatment condition but excluded from the sample, as is standard practice in a Randomized Clinical Trial.

**Fig. 2:**
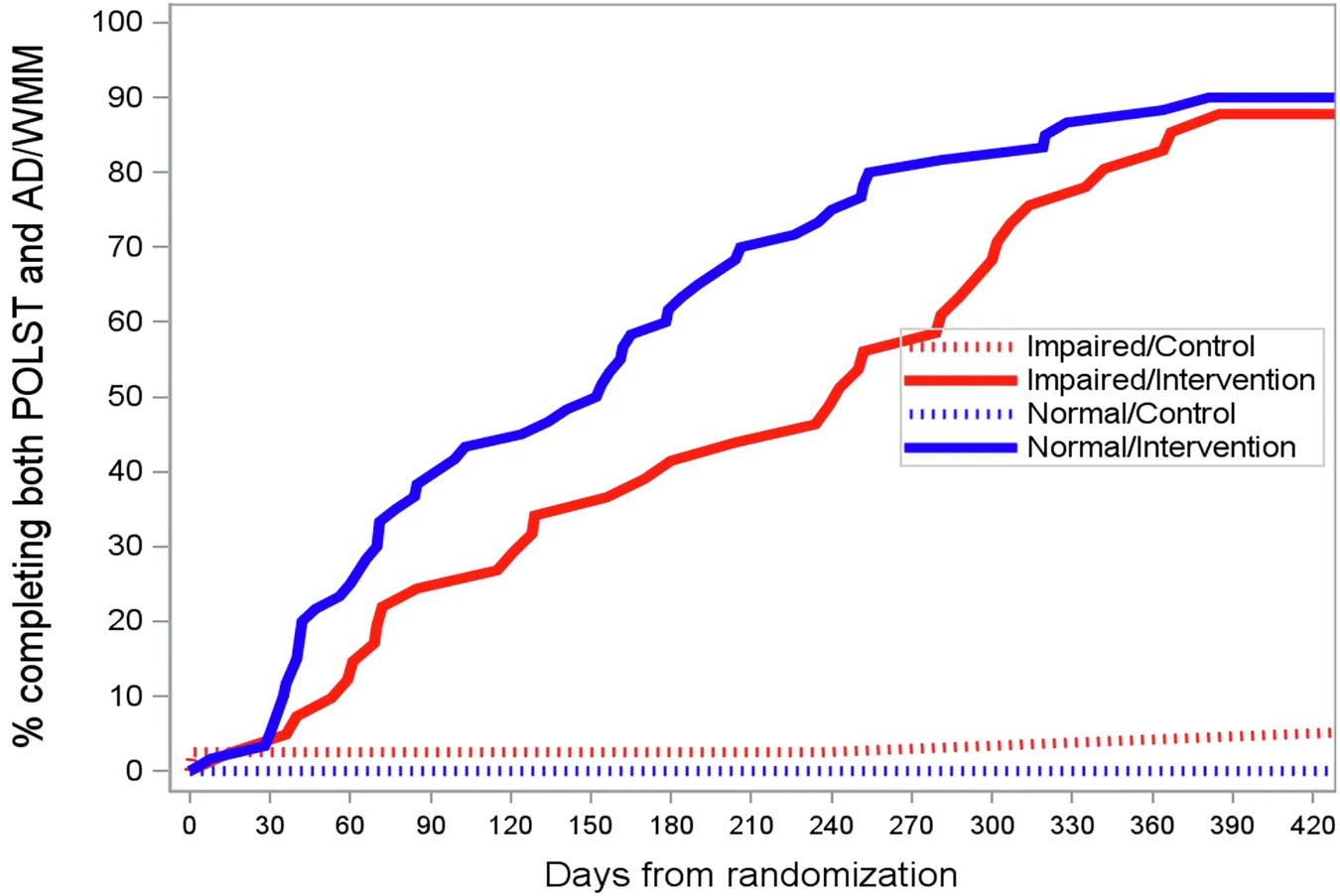
Time in Days to Completion and Documentation of Advance Care Planning. This figure shows the Kaplan Meier curves of time in days to completion and documentation of Advance Care Planning in the intervention and usual care arms. Participant groups had either CDR score = 0 or CDR = 0.5,1 or 2 at the time of study entry (baseline).

**Table 1:** Demographic characteristics and baseline scores for all study participants

CDR^a^ score at study entry	ELICIT Intervention + Usual Care		Usual Care	
	*CDR* = *0, n* = *60*	*CDR* = *0.5-2 n* = *41*	*CDR* = *0, n* = *59*	*CDR* = *0.5-2, n* = *40*
**Characteristics**	% or median (IQR) ^b^	% or median (IQR)	% or median (IQR)	% or median (IQR)
**Age**	75 years (70.5,80)	74 years (69,79)	72 years (69,80)	74 years (69.5,78)
**Charlson comorbidity rating**				
Moderate comorbidity	5.0	2.4	8.5	5.0
High Comorbidity	95.0	97.6	91.5	95.0
Gender				
Female	65.0	48.8	55.9	27.5
Male	35.0	51.2	44.1	72.5
**Education**				
HS^c^ Graduate/GED^d^	3.3	4.9	3.4	0.0
College	96.7	95.1	96.6	100.0
**Race/Ethnicity**				
White non-Hispanic	81.7	87.8	88.1	87.5
Other	18.3	12.0	10.2	12.5
Unknown	--	--	1.7	--
Hispanic/Latino	8.3	2.4	3.4	2.5
**Baseline scores on measures**				
ESAS^e^ score	6.5 (2.5,10)	10 (7,21)	6 (3,14)	10.5 (4.5,17.5)
PAM-13^f^ score	3.6 (3.2,3.8)	3.1 (2.9,3.5)	3.5 (3.3,3.9)	3.0 (2.7,3.3)
QOL-AD^g^ score	3.3 (3,3.7)	3.1 (2.9,3.4)	3.5 (3.1,3.8)	2.9 (2.7,3.2)

^a^Clinical Dementia Rating; ^b^Interquartile Range; ^c^High School; ^d^General Educational Development; ^e^Edmonton Symptom Assessment Scale; ^f^Patient Activation Measure (PAM); ^g^Quality of Life Alzheimer’s Disease (QOL-AD).

86.1% (87 out of 101) of patients in the intervention arm expressed palliative care needs compared to only 29.3% (29 of 99) in the usual care arm to the blinded evaluators. Seeking assistance with disease and symptom management was the most common primary palliative care need expressed by the patients followed by requests for assistance with care coordination. The median number of palliative care needs per patient in the intervention arm was 4 (IQR = 2, 6) and in the usual care arm was 0 (IQR = 0, 1). The difference between the two arms was highly significant with *p* < 0.0001, *χ*2 = 100.8 using the Mann Whitney test, with a large effect size (AUC = 0.90, SRD = 0.79).

There were three secondary outcomes analyzed using the Kruskal Wallis comparing the change scores from baseline to 12 months of the two arms. None were statistically significant. The ESAS change: Chi sq 3.8, *p* = 0.28; the PAM change: Chi sq 4.3, *p* = 0.0.23; the QOL-AD change: Chi sq 0.54, *p* = 0.91); degrees of freedom = 3.

However, at study baseline, cognitively impaired participants in both arms expressed significantly more symptom burden compared to the non-impaired with (a) worse Edmonton Symptom Assessment Scale (ESAS)^13^ total score at 10 (IQR 5,19) vs. 6 (IQR 3–11) for cognitively unimpaired patients; (b) worse Patient Activation Measure^14^ total score at 3.0 (IQR 2.8, 3.5) vs. 3.6 (IQR 3.3–3.8); and (c) Quality of Life^15^ score at 3 (IQR 2.7,3.3) vs. 3.4 (IQR 3.1–3.8).

Three people (1.5%) out of the 200 who enrolled chose to withdraw from the study. There were no adverse reactions reported or participant complaints during the study. In fact, 60 participants allocated to the intervention arm spontaneously expressed gratitude and positive feedback to the study team compared to three participants from the usual care arm.

Older adults with cognitive impairment are often excluded^16^ from randomized clinical trials. A recent scoping review^17^ identified that high methodological quality RCTs testing advance care planning are very sparse in PLWD. Our RCT successfully recruited 200 community-dwelling older adults with chronic illnesses, moderate to severe co-morbidities and varying levels of cognitive impairment. The baseline completion rate of the AD and POLST was very low for all study participants; for the usual care arm this remained largely unchanged over the entire study period (December 2019 through October 2024). This underscores that advance care planning completion would not have happened for these patients without the nurse-led intervention.

Older adults with cognitive impairment often lose their ability to participate in goals-of-care conversations and complete advance care planning. It is known that patient-designated and next-of-kin surrogates incorrectly predict the patient’s end-of-life treatment preferences^18^. There is a window of opportunity early in the dementia illness trajectory when the patient retains mental clarity and decisional capacity. Goals-of-care discussions and advance care planning should be documented in this window while patients are still able to voice their values and preferences for care, and in our study, the nurses were able to do this for patients in the intervention arm. Patients tend to choose less aggressive interventions when they participate in advance care planning^19,20^. While the patient’s goals and preferences may change over time, conducting a goals-of-care discussion and documenting advance care planning documents will serve to establish a baseline of their preferences while they still have the ability.

There is a growing shortage of the palliative care workforce. There is also an escalating need for early implementation of primary palliative care for chronically ill older adults in the community with dementia and other serious illnesses. The ELICIT model can benefit (a) seriously ill patients in the community as they will have earlier access to primary palliative care; (b) primary care providers as they will receive assistance from registered nurses in providing primary palliative care and (c) registered nurses who are interested in upskilling to provide primary palliative care as it will help them forge meaningful longitudinal relationships with chronically and seriously ill patients.

Our trial is notable for the following reasons: (a) To our knowledge, this is the first nurse-led, virtual intervention that provided early primary palliative care for patients. (b) As it is a virtual intervention, it is scalable. (c) This study has demonstrated the scope of nurses in providing primary palliative care.

This study has the following limitations: (a) The primary palliative care was provided by registered nurses. Registered nurses can assess patients and identify their medical and psycho-social needs, provide care coordination and education, and conduct goals-of-care conversations. However, nurses cannot independently make a diagnosis or prescribe medications. They worked with the patient’s PCP to fulfill all their medical needs. (b) The study population were mostly Non-Hispanic White and well educated and the intervention was conducted in English. We have translated our intervention into Spanish and pilot tested it in a small group of mono-lingual Spanish speakers. We are currently analyzing the results of the new study. (c) The long-term impact of the intervention is currently unknown. We are conducting long-term follow up of study participants to better understand if the intervention has any impact on their end-of-life care and whether they received goal-concordant care.

In this randomized clinical trial, a virtual, nurse-led, primary palliative care intervention resulted in identification of primary palliative care needs of our participants and in high rates of completion of goals-of-care discussions and advance care planning documentation. Future work should focus on evaluating the long-term impact of the ELICIT intervention on provision of goal-concordant care to older adults living with chronic and serious illnesses.

## Methods

### Study design and setting

Overview: ELICIT (*Early paLlIative Care IntervenTion*) is a 12-month, virtual, double-blinded, cognition-stratified RCT evaluating the impact of a {nurse-led, primary palliative care intervention plus usual care} compared to {usual care} on consenting chronically ill older adults living in the community. The trial protocol^14^ was reviewed and approved by the Stanford Institutional Review Board and registered with Clinicaltrials.gov (NCT03881579) on 2018-10-08. The consent form had the contact details for the Institutional Review Board so that participants could confidentially report any concerns or adverse effects from the study.

#### Participants, recruitment, and consent

Inclusion criteria: Older adults with chronic illnesses who had been seen by a neurologist and had undergone a full neurological assessment by the Stanford Alzheimer’s Disease Center multi-disciplinary team were eligible to participate if they were living in the community and had a clinical dementia rating (CDR)^15^ score between 0 and 2. The CDR is a cognition assessment system developed by the National Alzheimer’s Coordinating Center (NACC), and the scores range from 0 (normal cognition) 0.5 (Mild Cognitive Impairment or MCI), 1.0 (mild dementia), 2.0 (moderate dementia), and 3.0 (severe dementia). Participants were enrolled from December 2019 through April 2022.

Exclusion criteria: Participants with a CDR of 3 or living in an institutional setting were not eligible to enroll in the study.

Study participants were recruited using electronic flyers. Interested participants underwent a two-stage screening protocol. The initial screening was conducted by telephone to describe the study and assess if the participant was eligible and willing to participate. Participants who expressed interest in the study were scheduled for a video encounter where the research coordinators described the study details and answered any questions. If the participant was willing to enroll, the research coordinators video recorded a verbal consent. After obtaining consent, patients were randomized to the intervention or usual care arm. As we wished to compare the impact of the ELICIT intervention on the cohort of patients with CDR = 0, to those with CDR > 0, we powered the study adequately to allow comparison of two subgroups within the usual care and intervention arm. As this is a study of patients with cognitive impairment, the patient’s primary care partners were allowed to be present during the intervention as observers. In this paper, we focus on the impact on patients.

#### Randomization procedure

The RCT conformed to the Consolidated Standards of Reporting Trials (CONSORT) guideline. We randomized patients with CDR = 0 and those with CDR > 0 separately to the two treatment groups, employing the Efron randomization procedure to ensure balanced group sizes. The study biostatistician (HCK) provided oversight of the randomization protocol, and the study database manager (EN) did the computer-based randomization, and he was the only person who had access to the random sequence. Study evaluators, who were blinded to the treatment condition, assessed all study participants on study entry (month zero), month 4, and month 12. All participants were blinded to their study allocation arm. Only those participants who were randomly assigned to the intervention arm were seen by the study nurses as described below. As this was a virtual video intervention, technical support was provided to help participants log into the secure video link and participate in the encounter.

#### Intervention

The ELICIT intervention was delivered by trained study nurses to the patients in the intervention arm (n = 101). The intervention focused on the four primary palliative care domains within the current scope of a registered nurse: (1) assessing and assisting with palliation of distressing physical symptoms; (2) assessing and supporting emotional, social, cultural, and spiritual aspects of care; (3) establishing goals-of-care, identifying a surrogate decision maker, and completing of advance care planning documentation; and (4) providing care coordination support.

The intervention was virtual and delivered over a 12-month period and comprised of an initial nursing encounter session (S1) followed by 11 monthly follow-up encounters (S2-S12). The initial session (S1) was a one-hour video session. During this session, the nurses completed an assessment of the patient’s primary palliative care needs, initiated a goals-of-care conversation and introduced the concept of advance care planning. Scheduled follow up sessions (S2-S12) occurred monthly, lasted up to 30 minutes each, and were conducted as video visits except when the patient expressed preference for phone-based visits. After each session, the nurses discussed the patient’s needs with the study palliative care physician consultant (VSP) and crafted a care plan tailored to address the individual needs of each patient. For any medical needs identified in the care plan, the research nurses contacted the patient’s primary care physician (PCP) and worked with them to fulfill the patient’s needs during the 12-month intervention period. When patients expressed any urgent needs, the nurses helped them coordinate visits to urgent care services or the emergency room and notified their PCP after getting the patient’s permission. Requests for education about end-of-life issues, hospice care, and for community resources were fulfilled by the study nurses. After identifying the individual patient’s goals-of-care, the nurse used the Stanford Letter Project’s^17^ ‘What Matters Most Letter Advance Directive’ (AD) to help the patient document their wishes. The nurses educated the patients about the POLST form, reviewed the care choices and helped them complete it. Once the patient’s goals were clarified, the nurses notified each patient’s PCP. During their next clinical encounter, the PCP discussed and verified the patient’s goals-of-care and worked with them to complete and sign the POLST form (both the patient and their PCP must sign the POLST form). Once signed by the PCP, all patients were provided with a print and electronic copy of their signed and witnessed AD form and POLST form. The nurses also educated the patient and care partners about the correct usage of the Advance Care Planning documents (for example, to place the POLST form on their refrigerator door to be given to the emergency medical technicians in the event of a medical emergency).

For participants in both arms who expressed any palliative care needs to the blinded study evaluators during the evaluation visits, the study manager was notified immediately. For those in the intervention arm, the study manager conveyed the patients’ needs to the study nurses, who assessed and palliated these needs during the 12-month intervention period. For the patients in the usual care arm, for humanitarian reasons, the study manager contacted the primary care team and conveyed the patient’s requests after getting the patient's permission to do so.

#### Outcome measures

The primary outcome measures of the study were (a) time to completion of both the AD and POLST and uploading of the signed documents into the Electronic Health Records, and (b) the number of palliative care needs expressed to the evaluators by the patients over the 12-month period. The secondary outcome measures were score changes in the Edmonton Symptom Assessment Scale (ESAS), the Patient Activation Measure (PAM), and the Alzheimer’s Disease Quality of Life (ADQOL) measure at baseline and then again at months 4 and 12.

Edmonton Symptom Assessment Scale (ESAS): The ESAS is a brief and standard measure of symptom burden in life-limiting diseases, including dementia. It has been translated to Spanish and validated with an internal consistency (Cronbach’s Alpha was 0.75), concurrent validity with the Rotterdam Symptom Check List (Kappa until 0.66). Discriminant validity (Spearman) found significant differences (*p* < 0.001) in classification by Karnofsky. The average time to complete the was 5.5 min. ESAS scores will be used for Specific Aim 1 analyses.

Quality of Life Alzheimer’s disease (QOL-AD): QOL-AD: The QoL-AD is a valid and reliable scale that is brief (13 items), has good content validity, good criterion concurrent validity and construct validity. Interrater reliability was strong with all Cohen’s kappa values > 0.70. Internal consistency was good with a Cronbach’s alpha of 0.82. Some people with severe dementia and a Mini-Mental State Examination score as low as 3 were able to satisfactorily complete the QoL-AD. QOL-AD scores will be used for Specific Aim 1 analyses.

Patient Activation Measure (PAM): This is a 13-item valid and reliable measure with scores ranging from 0-100 and provides insight into possible strategies for supporting activation among participants with chronic illnesses at different points along the continuum.

#### Power calculations

For two primary hypotheses, with an overall 5% significance level, 80% power and a two-tailed test with equal number randomization, we determined that a total of 200 subjects: 100 for each arm (usual care and usual care plus nurse intervention) ensured adequate power for the two groups of patients (CDR = 0 and CDR > 0) within each arm. This sample size also allowed for later exploration of possible moderators and mediators of treatment effect.

#### Statistical analysis

All analyses of the RCT were performed using an intention-to-treat approach, i.e. all patients who were randomized were included in the analysis. For baseline characteristics, variables were described as medians and IQRs. A Cox Proportional Hazards Model was used to compare the time to completion of the advance care planning documents (advance directives and the POLST) and uploading them into the electronic health records. A Kruskal-Wallis test was used to compare the two RCT arms on the number of supportive care needs per patient reported to the evaluators over the 12-month period. For the two primary outcomes, statistical significance was defined at a 2-tailed with a significance level of 0.025 to ensure an overall error rate of less than 0.05. The secondary outcomes (ESAS, PAM, and Quality of Life measure score change) were evaluated by a linear mixed model for repeated measures, using a regression on {ln(*t* + 1)} and assuming auto-regressive error covariance structure.

## Supplementary information

supplementary_information

## Data Availability

The datasets generated and analyzed during the current study are not yet publicly available due to the fact that this is an active and ongoing study and we are still collecting data to be analyzed for the next phase of this project. Once the study has been completed, data will be available from the corresponding author on reasonable request.

## References

[1] 2024 Alzheimer’s disease facts and figures. *Alzheimers Dement*. **20**, 3708–3821 (2024). 10.1002/alz.13809 PMC11095490 38689398

[2] Malhi, R., McElveen, J. & O’Donnell, L. Palliative care of the patient with dementia. *Dela. J. Public Health* **7**, 92–98 (2021). 10.32481/djph.2021.09.012 PMC8482977 34604771

[3] Van der Roest, H. G. et al. Subjective needs of people with dementia: a review of the literature. *Int. Psychogeriatr.* **19**, 559–592 (2007). 10.1017/S1041610206004716 17201993

[4] de Sola-Smith, K. et al. Palliative care in early dementia. *J. Pain Symptom Manage.* **68**, e206–e227 (2024). 10.1016/j.jpainsymman.2024.05.028 PMC12060741 38848792

[5] Weisbrod, N. Primary palliative care in dementia. *Neurotherapeutics* **19**, 143–151 (2022). 10.1007/s13311-021-01171-x PMC8791082 35080735

[6] Ernecoff, N. C. et al. Comparing specialty and primary palliative care interventions: analysis of a systematic review. *J. Palliat. Med.* **23**, 389–396 (2020). 10.1089/jpm.2019.0349 PMC8255315 31644399

[7] Sleeman, K. E. et al. The escalating global burden of serious health-related suffering: projections to 2060 by world regions, age groups, and health conditions. *Lancet Glob. Health* **7**, e883–e892 (2019). 10.1016/S2214-109X(19)30172-X PMC6560023 31129125

[8] Kroenke, K. et al. Prevalence and predictors of symptoms in persons with advanced dementia living in the community. *J. Palliat. Med.* **25**, 1376–1385 (2022). 10.1089/jpm.2021.0402 PMC9492904 35357951

[9] Carmont, S. A. et al. Systematic review of the effectiveness, barriers and facilitators to general practitioner engagement with specialist secondary services in integrated palliative care. *BMJ Support. Palliat. Care* **8**, 385–399 (2018). 10.1136/bmjspcare-2016-001125 28196828

[10] Periyakoil, V. S., Von Gunten, C. & Kraemer, H. Virtual, nurse-led early primary palliative care intervention (ELICIT) for community-dwelling older adults with cognitive impairment: protocol for a randomized controlled trial. *JMIR Res. Protoc.* **14**, e75082 (2025). 10.2196/75082 PMC12731548 41442238

[11] Hughes, C. P., Berg, L., Danziger, W. L., Coben, L. A. & Martin, R. L. A new clinical scale for the staging of dementia. *Br. J. Psychiatry* **140**, 566–572 (1982). 10.1192/bjp.140.6.566 7104545

[12] Charlson, M. E., Pompei, P., Ales, K. L. & MacKenzie, C. R. A new method of classifying prognostic comorbidity in longitudinal studies: development and validation. *J. Chronic Dis.* **40**, 373–383 (1987). 10.1016/0021-9681(87)90171-8 3558716

[13] Nekolaichuk, C., Watanabe, S. & Beaumont, C. the edmonton symptom assessment system: a 15-year retrospective review of validation studies (1991–2006). *Palliat. Med.* **22**, 111–122 (2008). 10.1177/0269216307087659 18372376

[14] Hibbard, J. H., Stockard, J., Mahoney, E. R. & Tusler, M. Development of the patient activation measure (PAM): conceptualizing and measuring activation in patients and consumers. *Health Serv. Res.* **39**, 1005–1026 (2004). 10.1111/j.1475-6773.2004.00269.x PMC1361049 15230939

[15] Thorgrimsen, L. et al. Whose quality of life is it anyway? The validity and reliability of the Quality of Life-Alzheimer’s Disease (QoL-AD) scale. *Alzheimer Dis. Assoc. Disord.* **17**, 201–208 (2003). 10.1097/00002093-200310000-00002 14657783

[16] Mańko, A. et al. Exclusion of older adults from randomized controlled trials in rheumatoid arthritis. *Rheumatology (Oxford)* **63**, 672–679 (2024). 10.1093/rheumatology/kead257 37243691

[17] Giordano, A. et al. Advance care planning in neurodegenerative disorders: a scoping review. *Int. J. Environ. Res. Public Health* **19**, 803 (2022). 10.3390/ijerph19020803 PMC8775509 35055625

[18] Ayalon, L., Bachner, Y. G., Dwolatzky, T. & Heinik, J. Preferences for end-of-life treatment: concordance between older adults with dementia or mild cognitive impairment and their spouses. *Int. Psychogeriatr.* **24**, 1798–1804 (2012). 10.1017/S1041610212000877 22613082

[19] Schoenfeld, E. M. et al. Patient preferences regarding shared decision making in the emergency department: findings from a multisite survey. *Acad. Emerg. Med.* **25**, 1118–1128 (2018). 10.1111/acem.13499 PMC6185792 29897639

[20] Geerse, O. P. et al. Effects of shared decision making on distress and health care utilization among patients with lung cancer: a systematic review. *J. Pain Symptom Manage.* **56**, 975–987.e5 (2018). 10.1016/j.jpainsymman.2018.08.011 30145213

